# NIR-ViS-UV broadband absorption in ultrathin electrochemically-grown, graded index nanoporous platinum films

**DOI:** 10.1038/s41598-024-73204-2

**Published:** 2024-09-30

**Authors:** Sarmiza-Elena Stanca, Venkata R. Rayapati, Abhik Chakraborty, Jan Dellith, Wolfgang Fritzsche, Gabriel Zieger, Heidemarie Schmidt

**Affiliations:** 1 Leibniz Institute of Photonic Technology, Albert-Einstein-Straße 9, 07745 Jena, Germany; 2 Institute of Solid State Physics, Friedrich-Schiller- Universität Jena, Helmholtzweg 3, 07743 Jena, Germany

**Keywords:** Nanoporous platinum, NIR-ViS-UV optical constants, Graded index layer, Nanostructure-reflectivity relationship, Nanostructure-absorption relationship, Effective medium approximation, Materials science, Optics and photonics, Physics

## Abstract

Nanoporous platinum broadband absorber has attracted interest in thermosensorics and IR photodetection due to its unique properties. In this work we report the physical mechanism underlying broadband absorption in electrochemically-grown, nanoporous Pt films by analyzing NIR-ViS-UV spectral ellipsometry data of nanoporous Pt films in dependence on the Pt film thickness (27, 35, 38, 48 nm). For the two thinner films a single layer model with a graded optical index Pt surface layer was used. For the two thicker films a two-layer optical model with a constant optical index Pt substrate layer and a graded optical index Pt surface layer was used. The graded optical index of the Pt surface layer reduces surface reflectivity and the constant optical index Pt substrate layer supports multiple reflections in the Pt film. Finally, we relate the thickness dependent optical index with the nanostructure of the nanoporous Pt film, which can be controlled in the electrochemical growth process. We observed that while in the transverse plane the multilayer exhibits graded refractive index, in the top horizontal planes the multilayer assembly exhibits discontinuous refractive index values due to the distribution of platinum crystal islands in air, which allows a metamaterial behavior of the whole system.

**Supplementary Information:**

The online version contains supplementary material available at 10.1038/s41598-024-73204-2.

## Introduction

The realization of maximum NIR-IR broadband absorption by a surface coating is essential for the improvement of the performance of NIR-IR thermosensors and photodetectors. Recent progress in theoretical nanophotonics and nanofabrication, e.g. surface coatings made of single or stacking layers of dielectrics^1^, nano/microstructures^2,3^ or a mixture of both, has enabled more flexibility in design and fabrication of NIR-IR broadband absorber layers. In particular, the emergence of plasmonics and metasurfaces allows for the realization of broadband and angle-insensitive absorber layers at an order of magnitude thinner than the operational wavelengths^4–6^. Ideal surface coatings for thermosensors and photodetectors are coatings that combine broadband antireflection (to reduce reflection) and broadband absorption (to enhance absorption). Broadband absorption due to ohmic losses in surface coatings supports the light-to-heat conversion, e.g. in thermoelectric sensors. It has been shown that surface coatings with metallic nanoparticles and nanostructures can couple the light effectively into the surface coating and heat up under illumination due to ohmic losses in the metallic nanoparticles^2^. Broadband absorption from 200 to 2500 nm has been realized using ALD-grown Ag nanoparticles on silica scaffolds^7^. However, the growth of surface coatings needs approximate 2 h per ALD supercycle and a continuous substrate heating to 100°C. Also, high cost caused by an enormous range of expensive ALD equipment and both large area upscalability and small area local fabrication remain obstacles for future integration of ideal surface coatings into thermosensors and photodetectors. We have developed a cost-effective electrochemical growth to fabricate robust, ultrathin nanoporous Pt IR-NIR-ViS-UV broadband absorber films with the potential for both large areas up-scalability up to 6 inch (= 0.152 m) wafers and small area local fabrication down to few nanometers and with tremendously fast parameter screening possibilities of the electrochemical growth parameters, e.g. electrochemical bath, bias-time profile, substrate material and roughness^8–10^. The nanostructure of electrochemically-grown, nanoporous Pt films is thickness dependent. As a consequence, we expect that nanoporous Pt films reveal a graded refraction index. Recently, a graded index layer working in the 1500–200 nm bandwidths has been fabricated from Al-ZnS sawtooth-like and pyramid-like multilayers and the depth dependent optical constants have been analyzed using effective medium approximation^11^. A graded index layer for various bandwidths have been fabricated from SiON-SiO moth-eye-like nanopillars for 400–2500 nm bandwidth^12^, by coating silica pyramids with Ag nanoparticles for 220–2500 nm^7^, by stacking a MgF_2_-TiO_2_-Si-Ge-Cr multilayer on a glass substrate for 400–2000 nm^1^, from an Al_2_O_3_-SiO_2_ honeycomb-like structure on nanoporous SiO_2_ for 8000–15000 nm^2^, and from a Ti/Ge/Ti three-layer absorber for 8000–12000 nm^6^. All these works have in common that different subwavelength-structured materials form the gradient index layer in a bandwidth where the structured materials possess an optical contrast to the ambient material surrounding the structured material. We suggest analyzing the physical mechanism underlying broadband absorption in nanoporous metallic films using polarization dependent optical measurements, namely spectral ellipsometry measurements, on thin nanoporous metallic films where the optical contrast between the nanoporous metallic film and the ambient void is large. In addition, also the optical contrast between the substrate and the nanoporous metallic film plays a role by enhancing back reflection from the substrate into the nanoporous metallic film, thus enabling multiple reflections in the nanoporous metallic films. In this work we fabricated robust, ultrathin nanoporous Pt IR-NIR-ViS-UV broadband absorbers using electrochemical growth of Pt films, which is of high importance for local integration and the reduction of mass, fabrication time, and cost of the detector. We analyzed the relation between the thickness dependent structure and thickness dependent optical constants of the nanoporous Pt film. A Pt broad absorber needs a thickness of ca. 300 nm to reveal zero-valued reflectivity^2–4^. However, a rather thick nanoporous Pt film (300 nm) cannot be used in ellipsometry to analyse the optical constants of the nanoporous Pt film close to the substrate. Therefore, in this work we prepared ultrathin nanoporous Pt films of different thickness (27, 35, 38, 48 nm) using electrochemical growth. We determined the complex optical constants of the 4 different nanoporous Pt films measured by IR-NIR-ViS-UV spectral ellipsometry in dependence on the Pt film thickness. For the two thinner nanoporous Pt films (27 and 35 nm) we used a single layer model with a graded optical index Pt layer. For the two thicker nanoporous Pt films (38 and 48 nm) we used a two-layer optical model with a constant optical index Pt layer and a graded optical index Pt layer on top. In this work we use “Constant index Pt layer” and “substrate Pt layer” equivalently and we use “Graded index Pt layer” and “surface Pt layer” equivalently.

## Results

**Fig. 1:**
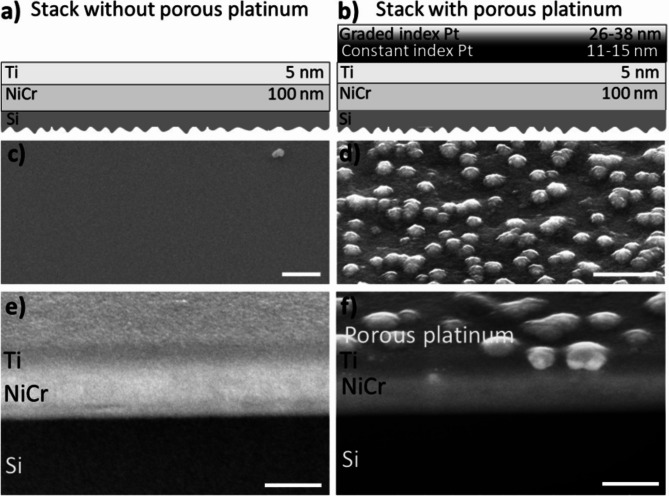
Stack without and with nanoporous platinum. (**a**, **b**): Schematic representation of the stack without (**a**) and with nanoporous platinum film (graded index Pt layer and constant index Pt layer) (**b**). (**c**), (**d**): Top view SEM of the stack without nanoporous platinum: Si/NiCr/Ti (**c**) and with nanoporous platinum: Si/NiCr/Ti/Pt constant index/Pt graded index (**d**); (**e**)-(**f**): Lateral view SEM of the stack without (**e**) and with (**f**) nanoporous platinum (graded and constant index Pt layer): Si/NiCr/Ti/Pt constant index/Pt graded index. Using spectral ellipsometry the graded index Pt layer is analysed as a graded effective medium consisting of nanocrystalline platinum and void (air). The constant index Pt layer is compact and also consists of nanocrystalline platinum. The graded index Pt layer has a large negative real part of dielectric constant ε1 and a large imaginary part of dielectric constant ε_2_. The nanoporous platinum layer causes the strong absorption of light due to the large ε_2_ of the graded index layer. Scale bar 100 nm.

**Fig. 2:**
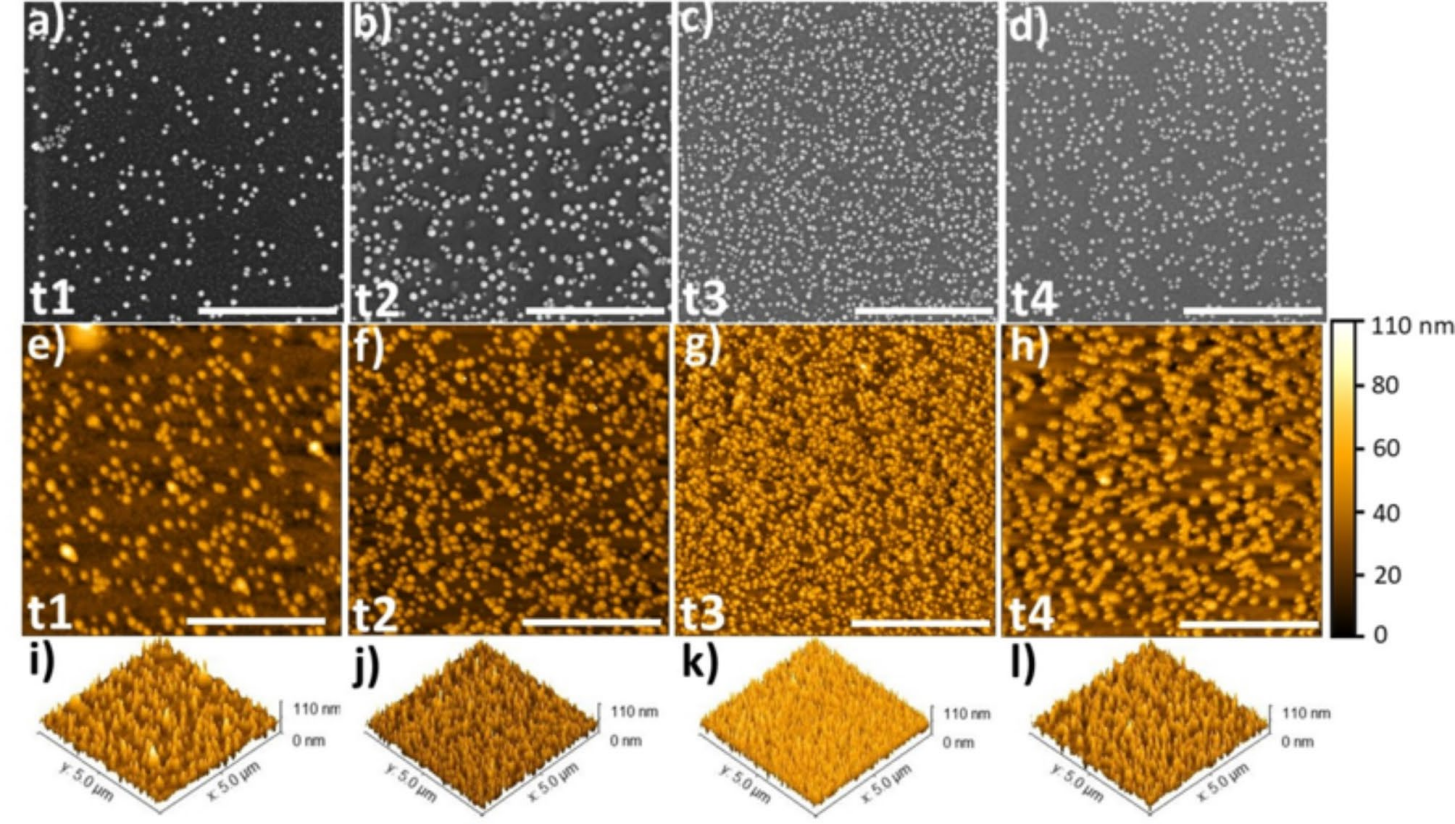
The morphology of the nanoporous platinum (graded and constant index Pt layer) electrochemically grown on Si/NiCr/Ti at four different electrolysis times (from left to right: t1 = 10s, t2 = 30s t3 = 40 s, t4 = 60 s). (**a**–**d**) Top view SEM images of the four samples which are labeled t1, t2, t3, and t4. (**e**–**l**) 2D (**e**–**h**) and 3D (**i**–**l**) height SPM image of the samples t1, t2, t3, and t4. The substrates for electrolysis of 0.5% PtCl_4_ are 1.5 cm x1.5 cm Si chips sputtered with highly reflecting 100 nm thick NiCr and 5 nm thick Ti layer. SEM and SPM scale bar: 2 μm.

**Fig. 3:**
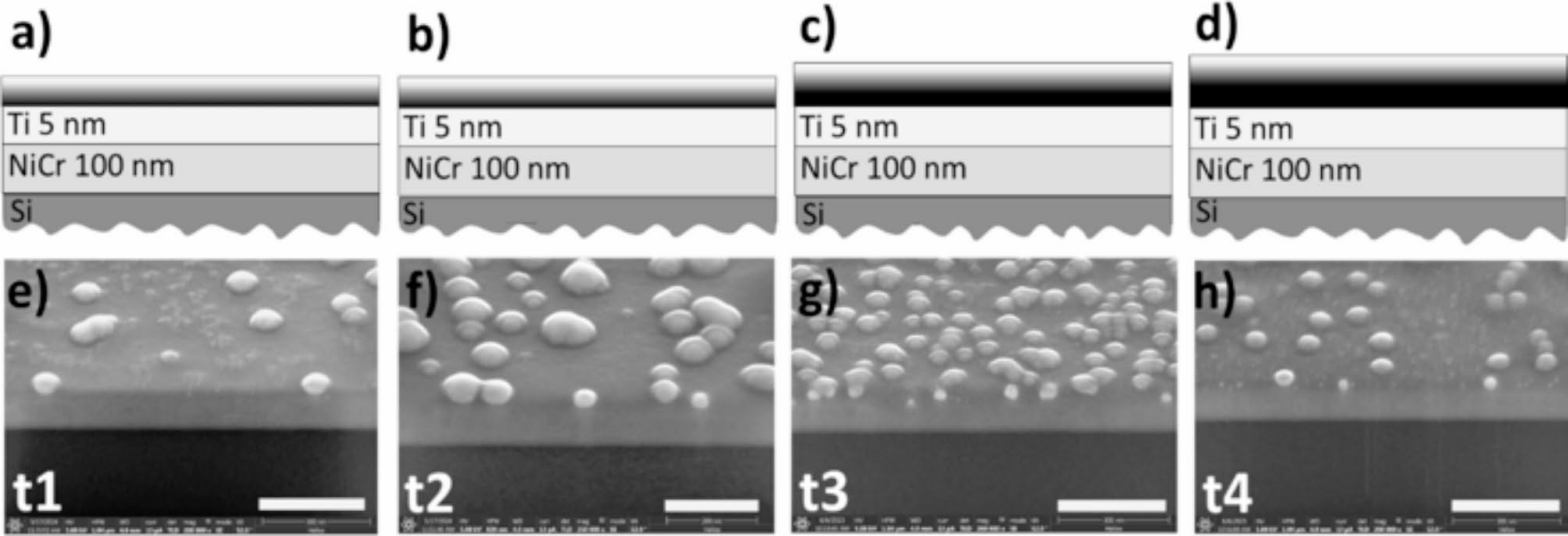
Cross-section of the samples t1, t2, t3, and t4 revealing the morphology of porous Pt layer. Schematic representation (**a**–**d**) and lateral SEM view on the morphology (**e**–**h**) of the four stacks (sample t1, t2, t3, and t4) with nanoporous platinum electrochemically grown on Si/NiCr/Ti at four different electrolysis times (10 s, 30 s, 40 s, 60 s). The scale bar is 300 nm.

In the following we report on the preparation of ultrathin nanoporous Pt films of different thickness (27, 35, 38, 48 nm) using electrochemical growth on Si/100 nmNi50Cr50/5nmTi with four different electrolysis times and labeled therefore t1, t2, t3, t4. Their optical and morphological properties have been studied by spectral ellipsometry (SE), Scanning Electron Microscopy (SEM), Scanning Probe microscopy (SPM) and Ulbricht-Sphere equipped Ultraviolet-Visible (UV-VIS) spectroscopy. Figure 1 shows the sketch and the SEM images of the nanolayer stack without/with nanoporous Pt films. The sketch indicates also the thickness of every layer including the thicknesses of graded index Pt layer and constant index Pt layer. Using spectral ellipsometry the graded index Pt layer is analysed as a graded effective medium consisting of nanocrystalline platinum and void (air). The complex optical constants are discussed equally either in terms of real part of dielectric constant ε_1_ (ε_1_ = n^2^–k^2^ < 0 or > 0) and imaginary part of dielectric constant ε_2_ (ε_2_ = 2⋅n⋅k > 0) or in terms of refractive index n and extinction coefficient k. The constant index Pt layer is compact and also consists of nanocrystalline platinum. The graded index Pt layer has a large negative ε_1_ and a large positive ε_2_. The morphology of the nanoporous platinum (graded and constant index Pt layers) is presented in Fig. 2. The top view SEM and SPM images of those electrochemically incomplete grown platinum layers show spherical shapes of the structures (Fig. 2a–l). These assemblies are made of metallic platinum crystals that provide a specific interaction with the light rays, displaying rather a trapping than a reflection of the light in certain wavelength regions. The SEM images of the FIB cut show the lateral view on the multilayer system (Fig. 3).

### Spectral ellipsometry

**Fig. 4:**
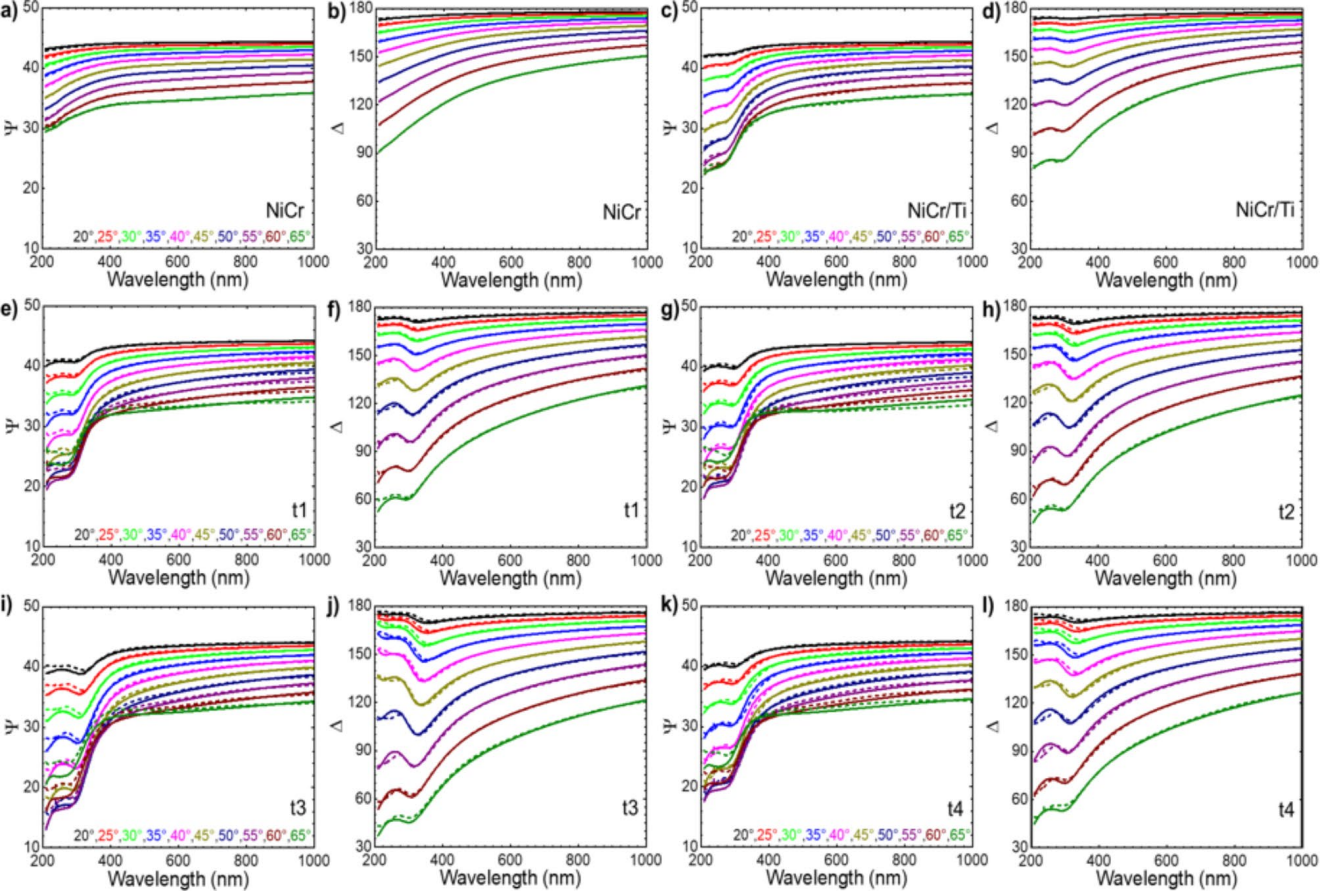
Experimental and modelled spectral ellipsometry parameters (**a**, **c**, **e**, **g**, **i**, **k**) Ψ and (**b**, **d**, **f**, **h**, **j**, **l**) Δ taken at 10 different angles of incidence from 20° to 65° in the spectral range from 210 to 1000 nm using a RC2 spectral ellipsometer from J.A. Woollam. Solid lines represent experimental and dashed lines represent modelled data. We used 1.5 × 1.5 cm^2^ large Si/100 nm NiCr (NiCr sample) and a Si/100 nm NiCr/5 nm Ti (NiCr/Ti sample) substrates for reference measurements. Mean square error (MSE) for fitted samples NiCr, NiCr/Ti, t1, t2, t3 and t4 are 0.078, 0.177, 0.676, 0.625, 0.772 and 0.801, respectively.

**Fig. 5:**
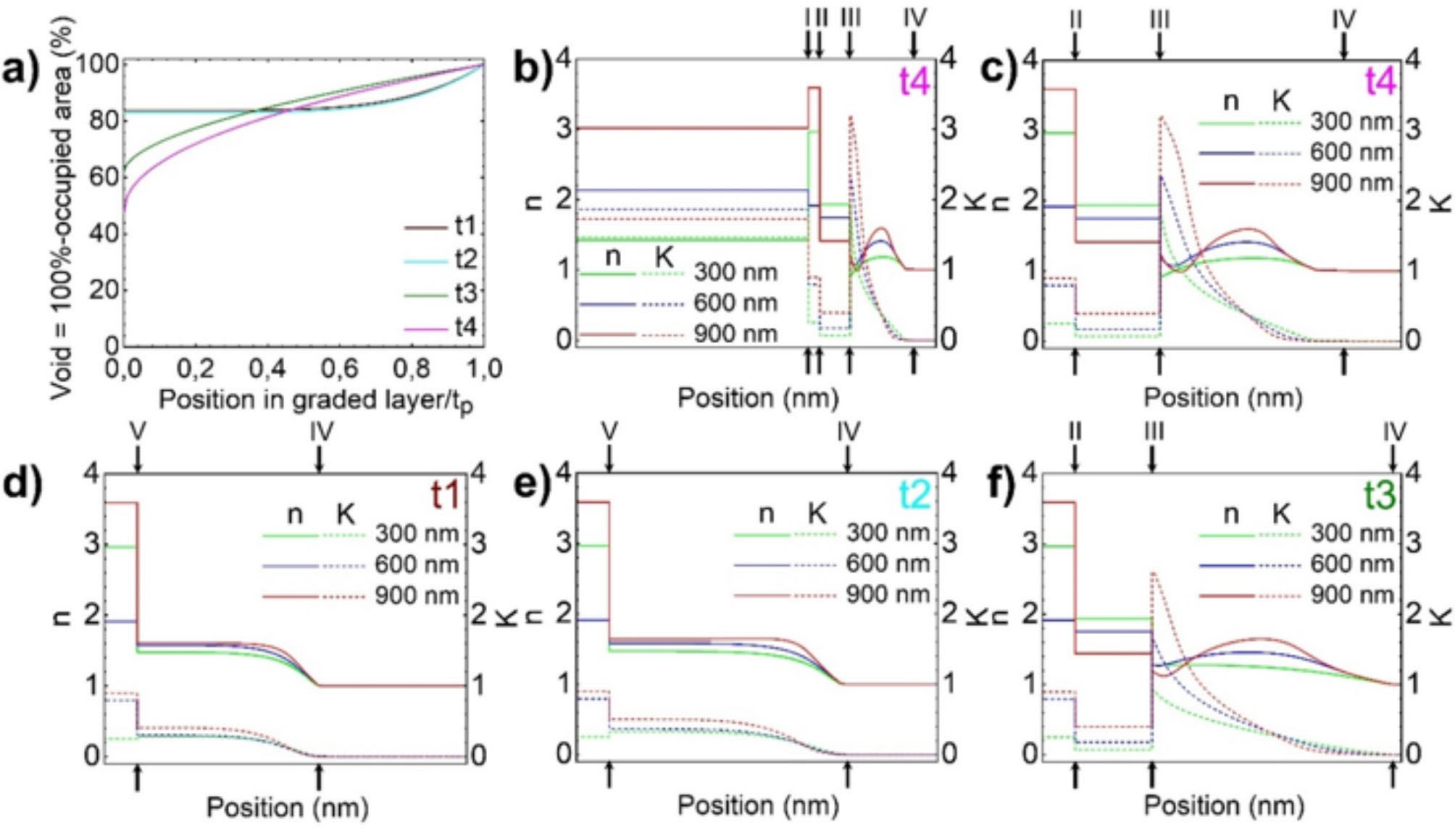
Profile of the graded index Pt layer in the samples t1, t2, t3, and t4 and depth dependent optical constants of those sample at 300 nm, 600 nm, and 900 nm. (**a**) Composition profile of graded index Pt layer vs. position in the graded index layer modelled using SE for sample t1, t2, t3 and t4. The position in the graded index Pt layer is normalized with respect to the thickness of the graded index Pt layer tp (Table 1). Refractive index n (solid line) and absorption index k (scattered line) at 300 nm (brown), 600 nm (green), and 900 nm (violet), across the (**b**) NiCr/Ti/Pt constant index/Pt graded index/void part of sample t4 and (**c**, **f**) across the Ti/Pt constant index/Pt graded index/Void part of samples t3 and t4. (**d**, **e**) Ti/ Pt graded index/Void part of samples t1 and t2. Position of interfaces NiCr/Ti (I), Ti/Constant index layer (II) and Ti/Graded index layer (V), Constant index/Graded index (III), and Graded index/Void (IV) is labeled with Roman numbers. Total length of x-axis is (**b**) and (**c**–**f**) 5 nm thick Ti layer is completely represented. Total length of x-axis in (**b**) is 155 nm and (**c**–**f**) is 55 nm. MSE for fitted samples NiCr, NiCr/Ti, t1, t2, t3 and t4 are 0.078, 0.177, 0.676, 0.625, 0.772 and 0.801, respectively.

**Fig. 6:**
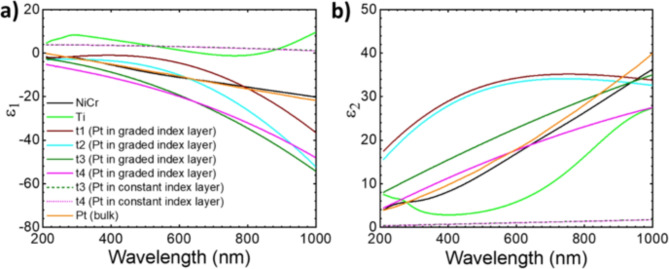
Optical constants (**a**) ε_1_ and (**b**) ε_2_ of Pt in graded index layer (t1, t2, t3, t4) and of Pt in constant index layer (t3 and t4), of NiCr in the NiCr sample, of Ti in the NiCr/Ti sample and of Pt (Bulk). Solid lines represent optical constants of nanocrystalline Pt in graded index Pt layer and dashed lines represent optical constants of nanocrystalline Pt in constant index Pt layer. Dark blue, light green, and light brown solid lines represent optical constants of NiCr in the NiCr sample, of Ti in the NiCr/Ti sample and of Pt (Bulk)^15^, respectively. MSE for fitted samples NiCr, NiCr/Ti, t1, t2, t3 and t4 are 0.078, 0.177, 0.676, 0.625, 0.772 and 0.801, respectively.

Variable angle spectroscopic ellipsometry (SE) in reflectance mode is used to investigate the optical properties of nanoporous platinum thin films (Figs. 4, 5 and 6). Spectroscopic ellipsometry is a non-destructive and an indirect method to measure the optical constants of thin films. It measures the ratio of amplitudes Ψ and the difference between phases Δ of ρ- and s- polarized incident light upon reflection^13^. A layered optical model is built to accurately represent the samples. Prior to the optical characterization of samples t1, t2, t3 and t4, reference samples of Si/100 nm NiCr (50:50) (named NiCr) and Si/100 nm NiCr (50:50)/5 nm Ti (named NiCr/Ti) are used to investigate the optical properties. The optical constants ε_1_, ε_2_ of reference samples are fixed for further modelling of samples t1, t2, t3 and t4. A B-spline model is used to model the optical constants of NiCr (50:50) thin film with initial material as Ni. A graded model with non-linear profile (Fig. 5a) has been used to model the graded index layer in the samples t1-t4 and a general oscillator model has been used to model the constant index layer in the samples t3 and t4. In a graded model, the total film thickness is divided into multiple sublayers (100) of equal thickness and a single Lorentz oscillator is used to model the samples. The Lorentzian line shape is mathematically defined as follows:

1 $$\:{\epsilon\:}_{2}=\frac{Amp.\:Br.\:{E}_{n}}{{E}_{n}^{2}-{E}^{2}-i.E.Br}$$

where $\:Amp$, $\:Br$ and $\:{E}_{n}$ represent the amplitude, Full-Width at Half-Maximum (FWHM), and centre energy of $\:{\epsilon\:}_{2}$, respectively. $\:{\epsilon\:}_{1}\:$is calculated from $\:{\epsilon\:}_{2}\:$by virtue of the Kramers–Kronig relationship. For Lorentzian oscillator, the FWHM is much smaller than the center energy. In a graded model the optical constants at any point are described as a mixture of the optical constants at the top and bottom of the film using Bruggeman Effective Medium Approximation (EMA). The Effective Medium Approximation (EMA) is used to calculate the optical constants of a mixed material. Calculations are based on combining two sets of optical constants together and the percentage of each material. The Bruggeman expression is given as^14^

2 $$\:{f}_{a}\frac{{\epsilon\:}_{a}-\epsilon\:}{{\epsilon\:}_{a}+2\epsilon\:}+{f}_{b}\frac{{\epsilon\:}_{b}-\epsilon\:}{{\epsilon\:}_{b}+2\epsilon\:}=0,$$

where $\:\epsilon\:$ is the effective dielectric function of the composite material. $\:{\epsilon\:}_{a}$ and $\:{\epsilon\:}_{b}$ are the dielectric functions of phases a and b in their pure forms. $\:{f}_{\left(a\:or\:b\right)}$ =$\:{n}_{\left(a\:or\:b\right)}$/($\:{n}_{a}+{n}_{b}$) is the volume fractions of the materials a and b. The graded model works by creating a series of thin homogenous layers with optical constants that slightly change in each layer. To create a model of nanoporous platinum layer, which enables the extrapolation of the optical constants from ellipsometric measurements, we have combined the morphological information from top view and 3D images (Fig. 2) with the SEM images of the transversal cut samples (Figs. 1e,f, and 3). The morphology parameters of nanoporous platinum (graded index Pt layer and constant index Pt layer) are presented in the Table 1, which compiles the SPM and SEM data used for spectroscopic ellipsometry model. Ψ (Fig. 4a,c,e,g,i,k) and Δ (Fig. 4b,d,f,h,j,l) of the measured (solid lines) and modelled (dashed lines) spectral ellipsometric parameters for samples NiCr, NiCr/Ti, t1, t2, t3 and t4 are shown in Fig. 4. Measured and modelled data are largely in good agreement and the mean square error for fitted samples NiCr, NiCr/Ti, t1, t2, t3 and t4 are 0.078, 0.177, 0.676, 0.625, 0.772 and 0.801, respectively.

Composition profile for graded index Pt layer of sample t1, t2, t3 and t4 are shown in Fig. 5a. The position in the Graded index Pt layer is normalized with respect to the thickness of the Graded index Pt layer t_p_ (Table 1). Depth profile of optical constants, refractive index n (solid line) and absorption index k (scattered line) at 300 nm (green), 600 nm (blue) and 900 nm (red) are shown in Fig. 5b–f for samples t1 (Fig. 5d), t2 (Fig. 5e), t3 (Fig. 5f) and t4 (Fig. 5b,c). Absorption index, which represents the imaginary component of the index of refraction, called also extinction coefficient k^13^, is given as follows:

3 $$\:\text{k}=\frac{\alpha\:\lambda\:}{4\pi\:n}$$

where $\:\alpha\:$ is the absorption coefficient, $\:\lambda\:$ is the wavelength and *n* is the refractive index. For air with *n* = 1, $\:\text{k}=\frac{\alpha\:\lambda\:}{4\pi\:}$ (3´)

**Table 1:**
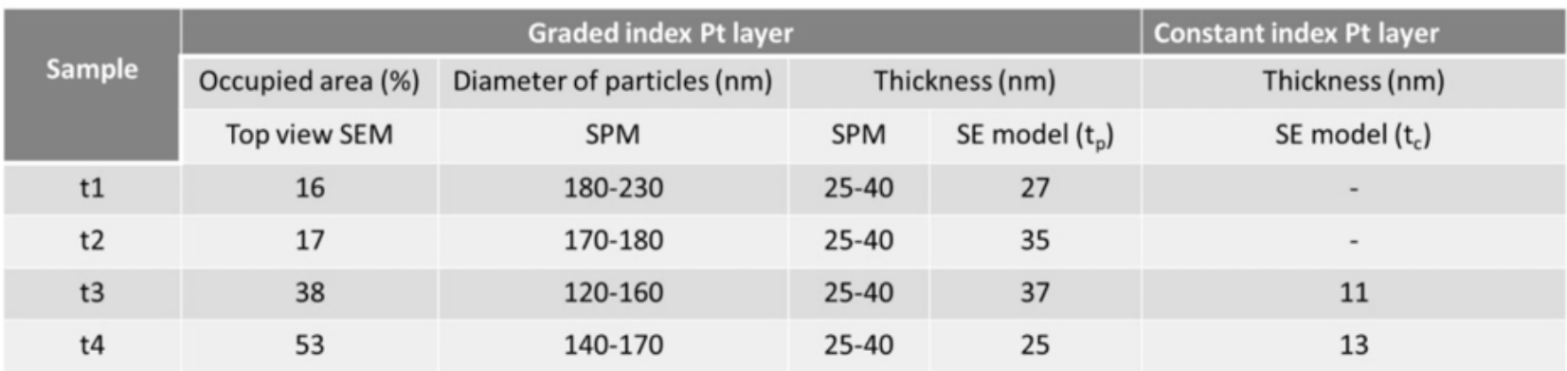
Morphology parameters of nanoporous platinum (graded index Pt layer and constant index Pt layer). Information on surface area covered by the particles (occupied area), diameter of particles in units of nm and thickness in units of nm of graded index layer has been extracted from Top view SEM, SPM, SPM/SE model, respectively. Information on thickness in units of nm of constant index Pt layer has been extracted from SE model.

Figure 5 shows the profile of the graded index Pt layer in the samples t1, t2, t3, and t4 and optical constants at three different wavelengths: 300 nm (brown), 600 nm (green), and 900 nm (violet). Roman numbers I, II, III, IV and V in Fig. 5b–f signify the following interfaces: (I) NiCr/Ti, (II) Ti/Constant index layer, (III) Constant index/Graded index, (IV) Graded index/Void and (V) Ti/Graded index layer. Optical constants n and k are constant for constant index layer, while for a graded index layer n and k changes with respect to the graded profile. Obtained thickness of graded index and constant index layers are tabulated in Table 1. The samples prepared at shorter electrolysis times (t1 and t2) exhibit an incomplete Pt layer. Therefore the optical constants change as a function of the investigated position as indicated in the Fig. 5d,e, with a smooth course to the value 1 at the interface IV (graded index Pt layer/void). This means to us that a low optical contrast at the interfaces will relay to an antireflection coating of the nanoporous platinum layer, while an accentuated optical contrast will conduct to an increase in absorption of the nanoporous platinum layer. Three important optical ingredients can be distinguished in the investigated multilayer system: (1) antireflection coating in the case of low optical contrast between the layers (graded index Pt layer), (2) absorption layer in the case of large optical contrast between the layers (constant index Pt layer), (3) back reflection from the first layer (Ti) of underlying substrates.

The modelling of measured ellipsometric data Ψ and Δ enables the estimation of the optical constants (ε_1_, ε_2_) of platinum in graded index Pt layer (solid lines) and in constant index Pt layer (dashed lines) (Fig. 6). Figure 6 shows the modelled optical constants of layers in the stack, ε_1_ (Fig. 6a) and ε_2_ (Fig. 6b) and a comparison of the ε_1_, ε_2_ of platinum in graded index layer and constant index layer (t3 and t4) with the ε_1_, ε_2_ of platinum bulk (orange colour) from data base^15^. The extracted ε_1_, ε_2_ shows negative real part ε_1_ in the measured/modelled visible wavelength region. The electric permittivity of the composite material is a function of every component. On one hand, Ti with its unstable electronic distribution on the orbitals (1s^2^2s^2^, 2s^2^2p^2^, 3s^2^4d^2^) has the tendency of electronic coupling, driving therefore the changes in electric permittivity of the surroundings. On the other hand, Ti due to its appropriate electric conductivity helps to produce a better growth of nanoporous platinum than NiCr^9^. To test the role of Ti, we investigated in a previous conducted work, two sets of stacks using spectroscopic ellipsometry, one set with no Ti in between NiCr and nanoporous platinum and the other with Ti in between NiCr and nanoporous Pt (Table S1, S2, Figure S1). The electric permittivity curves in Figure S1 suggest an enhanced precision and determinism in fabrication for the stack 2 (with Ti) compared to stack 1 (without Ti). The Ti underlayer seems to play an important role in the realization of the broadband absorber nanoporous Pt film. The light reflected back by this Ti layer into the nanoporous Pt film will be mainly absorbed by the graded index Pt layer (Fig. 5).

## Discussions

In this work we analyzed the physical mechanism underlying broadband absorption in nanoporous metallic films using polarization dependent optical measurements, e.g. spectral ellipsometry, on thin nanoporous metallic films where the optical contrast between the substrate and the nanoporous metallic film plays a role and where multiple reflections in the nanoporous metallic films can be encountered. Our approach considers a general, time-dependent electrochemically growth of the platinum nanocrystals, randomly distributed in a two-dimensional (2D) space, during the formation of the nanoporous platinum films. The ellipsometric model reflects the correlation of the occupied area and graded profile of nanocrystalline platinum (Fig. 4) with the four successively electrolysis times (t1, t2, t3, t4), started from unclosed films (t1, t2) to closed films (t3, t4) (Table 1; Figs. 3 and 4). Based on the SE model, the analysis of the ellipsometric data (Fig. 4) recorded for the samples t1–t4 for measured angles between 20°–65° with a step of 5° permits the determine the optical constants ε_1_, ε_2_ of graded index Pt layers (Fig. 5) and constant index Pt layers (Table 1). The optical constants of the other layers in the stack (Si, NiCr, Ti) have been extracted and displayed in the Figs. 5 and 6. In the 200–1000 nm wavelength region, the samples t3-t4 clearly exhibit negative values of the epsilon 1 in the graded layer model from − 20 at 200 nm to -60 (t3) and − 80 (t4) at 1000 nm (Fig. 6a), even if the estimated values of the ε for the nanocrystalline. Compared to this, the samples prepared at short electrolysis times t1, t2 exhibit less negative ε_1_, from − 1(t1, t2) at 505 nm to -20 (t1), and − 50 (t2) at 1000 nm and positive values for the wavelength interval 200–500 nm. The estimated ε_1_ for the closed platinum layer nanocrystalline platinum (t3, t4) indicated with dashed line in the Fig. 5a attains oscillating positive values. Si substrate shifts its epsilon values from − 20 at 300 nm to 45 at 400 nm being almost constant to 18 in the wavelength interval 500–1000 nm (Fig. 6a). The 100 nm NiCr (50:50) on top of Si substrate modified consistently the epsilon values (0 at 200 nm, and − 10 at 1000 nm). Adding the 5 nm Ti on the NiCr metallic layer leads to an increase of epsilon to 0 value almost in whole wavelength interval of interest (Fig. 6a).

The effective magnetic permeability and effective electric permittivity should be both considered to describe the composite interaction with the electromagnetic field. For the magnetic metallic films, where the near-field magnetic effects appear in the adjacent nanostructures, the estimation of optical constants by using only electric effective permittivity is insufficient. However, Pt and Ti are not magnetic materials. Thin films made of Ni-Cr and Co-Cr alloys, which were galvanically deposited from aqueous solutions with trivalent chromium ions and glycine, have been reported to have soft magnetic properties^16^. Therefore, at the interaction with light of our system, which has a diamagnetic nanoporous platinum on the top, one expects electric near field effects rather than magnetic near field effects. Nevertheless, for a more accurate approximation of the optical constants, an effective magnetic permeability along with the electric permittivity should be considered. However, there is no analytical model that can accurately describe the nanoporous irregular geometry. To understand the effective dielectric behavior, one can assume that the metallic nanostructure is a dipole that interacts with an external electromagnetic field^17–20^. Though, the vector potential associated with the electric and magnetic fields from the composite unit could only be defined by an exact geometry^19^, and not by an irregular structure as nanoporous platinum. The equation of expressing effective electric permittivity (ε_eff_) and effective magnetic permeability (µ_eff_)^19^ were applied to a precise geometry of the nanostructure, with a distance between the structures maintained constant, and higher order modes neglected. It would be of interest, the representation of the effective magnetic permeability (µ_eff_) for the t1–t4 samples, however the limitations caused by high order modes and randomly distribution of the platinum nanostructures impede a reliable representation of µ_eff_.

The optical constants data analysis focuses on quantifying how well the data generated by the optical model matches the measured data. The Complete Ease software^21^, which is used to process the ellipsometry data provides the mean squared error (MSE)^22^ values for the applied models.

The MSE is defined as a function of Ψ,Δ as follows^23^:

$$MSE=\sqrt {\frac{1}{{2 N - M}}\sum\limits_{{i=1}}^{N} {\left[ {{{\left( {\frac{{\psi _{i}^{{\bmod }} - \psi _{i}^{{\exp }}}}{{\sigma _{{\psi ,i}}^{{\exp }}}}} \right)}^2}+{{\left( {\frac{{\Delta _{i}^{{\bmod }} - \Delta _{i}^{{\exp }}}}{{\sigma _{{\Delta ,i}}^{{\exp }}}}} \right)}^2}} \right]} } =\sqrt {\frac{1}{{2 N - M}}{\chi ^2}}$$

where N is the number of (Ψ,Δ) pairs, M is the number of variable parameters in the model, and σ are the standard deviations on the experimental data points,

or it is defined as a function of N = Cos(2Ψ), C = Sin(2Ψ)Cos(Δ), S = Sin(2Ψ)Sin(Δ)^23^:

$$MS{E_{NCS}}=\sqrt {\frac{1}{{3n - m}}\sum\limits_{{i=1}}^{n} {\left[ {{{\left( {\frac{{{N_{{E_i}}} - {N_{{G_i}}}}}{{0.001}}} \right)}^2}+{{\left( {\frac{{{C_{{E_i}}} - {C_{{G_i}}}}}{{0.001}}} \right)}^2}+\left( {\frac{{{S_{{E_i}}} - {S_{{G_i}}}}}{{0.001}}} \right)} \right]} }$$

where “n” is the number of wavelengths, “m” is the number of fit parameters.

The MSE sums the differences between the measured data and model generated data over all the measurement wavelengths. The lower the MSE value, the better the fit between the measured and modelled data^21^.

**Fig. 7:**
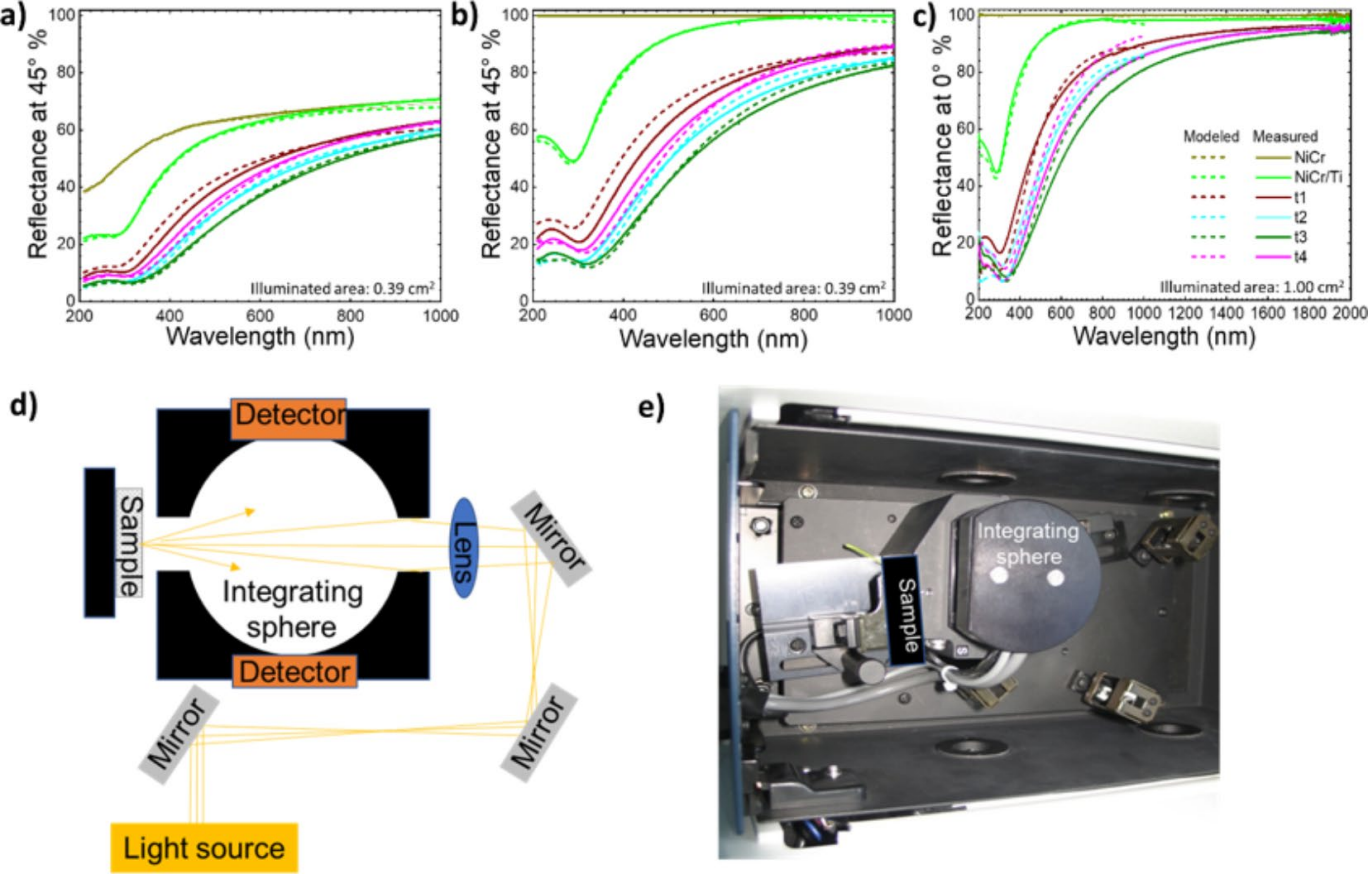
Measured (solid lines) versus modelled (dashed lines) reflectance of the stack with constant index Pt layer and graded index Pt layer. Reflectance at 45° angle of incidence (**a**–**b**) has been measured using spectral ellipsometry. Reflectance at normal incidence (**c**) has been measured using Ulbricht sphere. The reflectance data in (**b**–**c**) are normalized with respect to reflectance of the NiCr sample. Dashed lines represent the modelled reflectance using SE software and the optical constants shown in Fig. 6 (**d**–**e**) The scheme (**d**) and the photograph (**e**) of the equipment used to record total reflectance (specular and diffuse reflectance) using an integrating sphere setup.

**Fig. 8:**
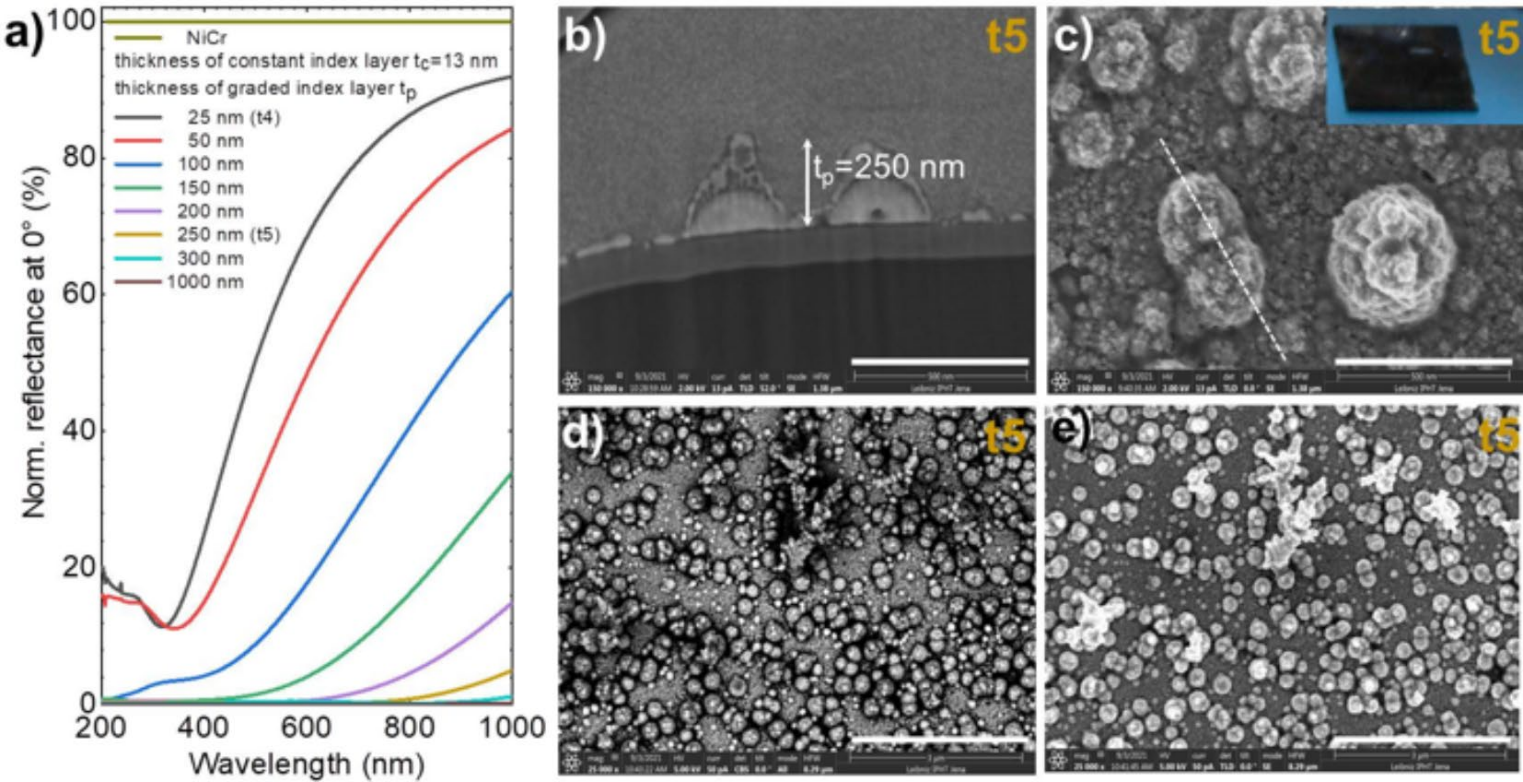
Modelled reflectance and SEM images of broadband absorbers with thick nanoporous Pt layer. Modelled reflectance of broadband absorbers decreases with increasing thickness of nanoporous Pt layer. (**a**) Modelled reflectance at normal incidence normalized with respect to NiCr layer. The thickness of the nanoporous platinum has been varied from 26 to 1000 nm. Optical constants of graded index and constant index layer have been taken from sample t4. Thickness of constant index Pt layer was also taken from sample t4. As for sample t1, t2, t3, and t4 the graded index layer was modelled as effective medium with a gradual change of optical constants (same change as in sample t4 with 47% void at constant index/graded index interface and 100% void at Graded index/Void interface, Fig. 5). (**b**–**e**) FIB (**b**) and SEM (**c**–**e**) of the thick nanoporous platinum layer (t5). The inset of the panel (**c**) represents the photograph of the sample t5 (1.5cmx1.5 cm). The panels (**c**–**e**) are top-view SEM images recorded in the same area of the sample with a ca. 250 nm thick nanoporous Pt layer. The white coloured domains of the backscattered electrons in the back-scattering electron (BSE) micrograph in panel (**d**) indicate occurrence of high atomic number elements, here of Pt atoms in the nanocrystals in constant index and graded index Pt layer.

We focused our work on elucidating the absorption rather than the anti-reflection phenomenon in porous platinum, therefore our experiments of recording the reflectance were conducted using the integrating sphere equipment. The measured reflectance spectra of the samples t1–t4 normalized to the NiCr mirror (black line) compared to the modelled reflectance spectra (dashed lines) are displayed in the Fig. 7. Even though, the multilayer system samples show a metallic reflectance, there is a wavelength region were the reflectance decreases at the same level exhibited by the sample of black nanoporous platinum^8–10^. The minimum reflectance peak at 300 nm of the substrate NiCr/Ti without nanoporous platinum slowly shifts to the 325 nm in the multilayer system with nanoporous platinum samples t1–t4 and the minima of the reflectance correspondingly decreases. The modelled reflectance of the nanoporous platinum grown on NiCr/Ti at gradually increased thickness from 33 to 300 nm shows a clear tendency of decrease (Fig. 8). There is a resonance wavelength shift from 325 nm (33 nm, 50 nm thickness) to 400 nm (100 nm thickness) along with a decrease in reflectance values.

From the diagrams in Fig. 7 one can predict closed to zero values for the reflectance at larger thickness of nanoporous platinum layer. Between the spectra of the nanoporous platinum films at different thicknesses, it is remarked the effect of slightly enlargement of the minima and decreasing the reflectance values by the increase of nanoporous platinum thickness. To estimate the reflectance at larger thickness, we modelled reflectance of broadband absorbers with thick porous Pt layer, imaged in the Fig. 8. Modelled reflectance of broadband absorbers decreases with increasing thickness of nanoporous Pt layer (Fig. 8a). This model predicts broadband spectra with close to zero reflection of nanoporous platinum at the thickness larger than 300 nm. There are interesting scientific approaches of ultrathin layer absorbers, which make use of doping an ultrathin silicon film^24^ or of spirally structure poly methylmethacrylate (PMMA)^25^, or of combining noble nanostructures with certain established oxides, Indium-Tin-oxide^26^, or taking advantage of the in-plane birefringence initiating by α-MoO_3_^27^. All these approaches attained subwavelength thickness (~ nm) into a bi- or multilayer architectures. We opted for the noble material, because it confers long term chemical stability and robustness. Our approach of one step electrochemical fabrication of the thin layers requires a small amount of noble salt, which also enables a relative accessible low-cost process. Therefore, compared with other materials, nanoporous platinum remains attractive of both fabrication cost, and optical performance and stability point of views. Organic absorbers are interesting of optical performance point of view however they are instable at high temperature, instead platinum exhibits a good thermal and chemical stability.

In summary, it became possible to fabricate robust, ultrathin nanoporous Pt IR-NIR-ViS-UV broadband absorber films using electrochemical growth, which is of high importance for local integration and the reduction of mass and cost of detector. We show that nanoporous platinum layers composed of constant index Pt layer and graded index Pt layer, electrochemically grown on Si/ NiCr/Ti substrates exhibit negative real part of permittivity in the visible wavelength region. Our findings predict a close to zero reflectance of the nanoporous platinum at a larger than 300 nm thickness of the nanoporous platinum layer in the ultraviolet, visible and mid infrared wavelength region. Moreover, we observed that in the perpendicular to the substrate plans, the multilayer exhibits graded refractive index values, while in the top horizontal planes parallel with the substrate, the multilayer assembly exhibits discontinuous refractive index values. As a result, the entire system exhibits metamaterial properties.

## Methods

### Multilayer substrate

A multilayer substrate (Si, NiCr, Ti) was constructed on silicon chips (1.5 cm × 1.5 cm) by sputtering them with 100 nm NiCr 50:50 and then with 5 nm Ti.

### Sputtering method

NiCr 50:50 nanolayers of 100 nm thicknesses and also Ti nanolayers of 5 nm thickness were deposited on silicon wafer substrates by sputtering (Sputtering System II, BesTec, Berlin, Germany) in the clean room at the Leibniz Institute of Photonic Technology Jena. Si wafer has been cleaned by Ar before NiCr deposition.

### Electrochemical growth of nanoporous platinum

Nanoporous platinum layer was electrochemically grown on the above obtained substrates from a 0.05% PtCl_4_ solution in isopropanol at lower concentration than described in our previous work^8–10^ at different electrolysis times (t1, t2, t3, t4). The investigated series are: Si/100nmNiCr/5nmTi, and Si/100nmNiCr/5nmTi/40nm nanoporous platinum.

### Ellipsometry

Spectral ellipsometry measurements were performed using J.A. Woollam RC2-U Variable Angle Spectral Ellipsometry (VASE) in reflectance mode. Ellipsometric reflectance, Ψ and Δ were acquired at different angles of incidence (20°–65° step length 5°) over a spectral range of 210–1000 nm with an initial alignment at 45°. Multiple angles and wavelengths were fit simultaneously in the optical model. Optical modeling and data analysis were done using CompleteEASE software. B-spline model with initial material as Ni is used to model 100 nm NiCr layer, Lorentzian oscillators are used to model 5 nm Ti layer, GenOsc model with Lorentzian oscillator is used to model the constant index layer and non-linear graded layer model with EMA approximation is used to model graded index layer. The graded layer model is considered with a graded profile (shown in Fig. 5a). Multi sample Fit is used for Sample t1 and t2 together and sample t3 and t4 together. Modelled optical constants from sample t1 is used as a reference for modelling the sample t2, t3, t4 for graded index and constant index Pt layers. Measured and fitted Ψ, Δ are shown in Fig. 4.

### UV-Vis-NIR-MIR spectroscopy

UV-Vis spectra were obtained with a Jasco V-670 spectrometer (Hachioji, Tokyo, Japan) using a 60 mm integrating sphere ISN-723/B008461118.

The MIR spectra were measured in a FTIR-Spectrometer (Bruker Instrument). The spectra were recorded with a resolution of 2 cm^−1^ in the spectral range 7500–500 cm^−1^. The samples were illuminated with a MIR light source and the spectra were collected with a standard FTIR detector with Mercury Cadmium Telluride (MCT) diode (D*:>2 × 10^10 ^cm Hz^1/2 ^W^−1^) liquid nitrogen cooled or with Deuterated L-alanine Triglycine Sulphate (DLaTGS) room temperature operated (D*:>2.7 × 10^8 ^cm Hz^1/2 ^W^−1^). The measurements were performed at the source aperture, collection mirror velocity and angle that show minimal noise.

### Scanning electron microscopy (SEM)

SEM measurements were performed with a field emission microscope JSM-6700 F (JEOL, Tokyo, Japan) and FEI Helios NanoLab G3 UC (ThermoFisher, The Netheerland). The energy of the exciting electrons was typically 5 keV. In order to enhance the surface sensitivity and in this manner the topographical impression some of the micrographs were taken at a stage tilt of 45°. Beside the detector for secondary electrons (SEI), Everhart-Thornley type, the system is equipped with different detector types (semiconductor and YAG type) for backscattered electrons.

### Focused ion beam (FIB)

For cutting and immediately imaging the layers, we used FEI-Helios NanoLab G3 UC (Thermo Fisher Scientific, Nederland) dual-beam instrument, which combines a focused ion beam (FIB) column with a high-resolution field emission (Schottky Thermal Field Emitter) scanning electron microscope (SEM). Retractable detectors for high contrast back scattered electrons BSE imaging are available in this instrument.

### Scanning probe microscopy (SPM)

The topography of the samples t1–t4 was investigated using a Dimension Edge Scanning Probe Microscope (SPM) (Bruker nano GmbH, Nano Surface Division, Karlsruhe, Germany) suitable for large samples up to 150 mm. The vertical noise floor is less than 50 pm RMS in appropriate environment. The imaging was performed in the tapping mode using an Al coated Si_3_N_4_ Budget Sensor cantilevers with a resonance frequency of approximate 300 kHz (Tap300).

## Electronic supplementary material

Below is the link to the electronic supplementary material.

Supplementary Material 1

## Data Availability

The datasets generated and analyzed during the current study are available from the corresponding authors on reasonable request.
