# Supplementary material for: NIR-ViS-UV broadband absorption in ultrathin electrochemically-grown, graded index nanoporous platinum films
Source: Sci Rep. 2024 Sep 30;14:22709. doi: 10.1038/s41598-024-73204-2 (PMC11442651; doi:10.1038/s41598-024-73204-2)
Supplement: Supplementary file 1 — Supplementary Material 1 [file 41598_2024_73204_MOESM1_ESM.docx]

**Supplementary Information**

**NIR-ViS-UV broadband absorption in ultrathin electrochemically-grown, graded index nanoporous platinum films**

Sarmiza-Elena Stanca^1*^, Venkata R. Rayapati^1,2^ **^*^,** Abhik Chakraborty^1,2^, Jan Dellith^1^, Wolfgang Fritzsche^1^, Gabriel Zieger^1^, Heidemarie Schmidt^1,2^**^*^**

^1^Leibniz Institute of Photonic Technology, Albert-Einstein-Straße 9, 07745 Jena, Germany

^2^Institute of Solid State Physics, Friedrich-Schiller- Universität Jena, Helmholtzweg 3, 07743 Jena, Germany

[*To whom correspondence should be addressed [heidemarie.schmidt@leibniz-ipht.de](mailto:heidemarie.schmidt@leibniz-ipht.de); [VenkataRao.Rayapati@leibniz-ipht.de](mailto:VenkataRao.Rayapati@leibniz-ipht.de); [sarmiza.stanca@leibniz-ipht.de](mailto:sarmiza.stanca@leibniz-ipht.de)]


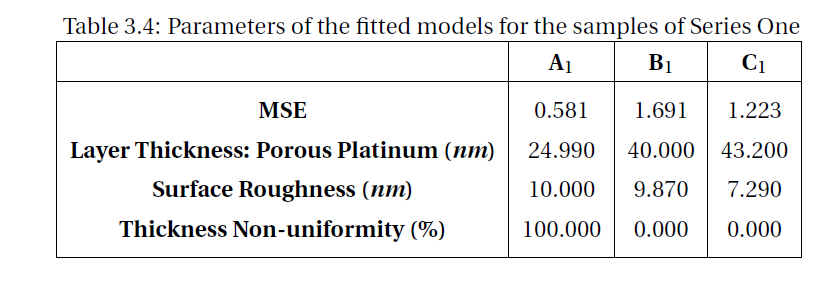
**Table S1**. Parameters of the fitted models for the platinum layers in the samples of stack 1 Pt 24,9nm Pt 40.0nm Pt 43.0nm

**Table S2**. Parameters of the fitted models for the platinum layers in the five samples of stack 2


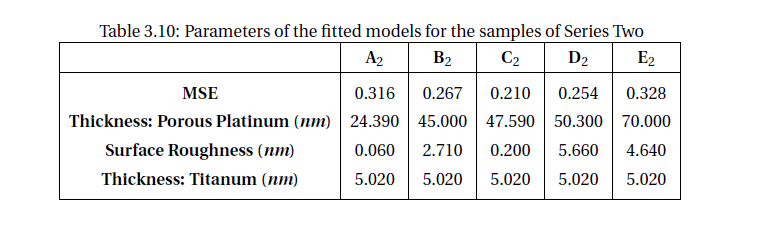
Pt24.3nm Pt45.0nm Pt47.6nm Pt50.3nm Pt70.0nm


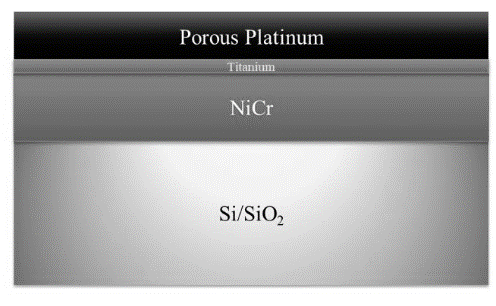

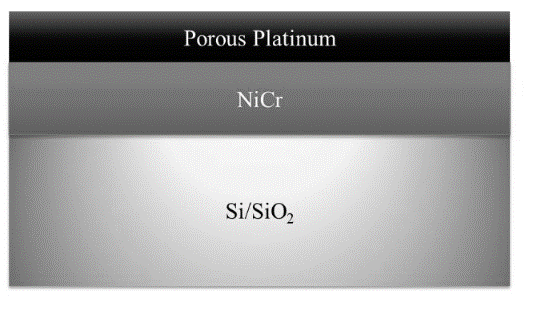
 Stack 1 Stack 2


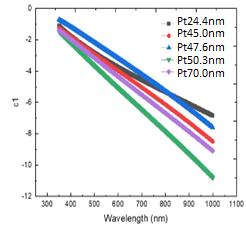

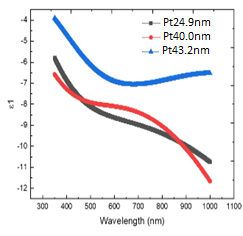


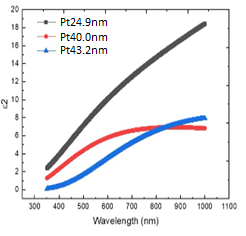

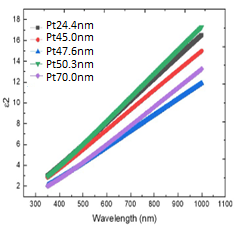


**Figure S1.** Schematic and modelled $\varepsilon$1 and $\varepsilon$2 of the samples in stack 1 and stack 2 using spectroscopic ellipsometry

Table S1 and table S2 indicate the fitted model parameters of the porous platinum layers in the samples of stack1 and stack 2. The porous platinum layer has been deposited over the layer of NiCr in stack 1 and over ultrathin layer of titanium like in the stack 2. The modelled roughness for samples in stack 2 is lower than for the samples in stack 1. Furthermore, the modelled permittivity for samples in stack 2 exhibits higher predictability in comparison to the samples in stack 1. This reinforces our conclusion that deposition of porous platinum layer over ultrathin layers of titanium and NiCr is more suitable for reliable ellipsometric study compared to the samples without Ti. The modelled electric permittivity curves in figure S1 suggest an enhanced precision and determinism in fabrication for the stack 2 (with Ti) compared to stack 1 (without Ti).
